# Impact of Experimental Congenital Toxoplasmosis on the Thyroid Gland: Histopathological and Immunobiochemical Indices Assessment

**DOI:** 10.1007/s11686-024-00969-x

**Published:** 2025-01-24

**Authors:** Hanan Abd Elgawad, Manar S. Elmehankar, Nairmen Nabih, Heba Sheta, Soha I. Awad

**Affiliations:** 1 Department of Medical Parasitology, Faculty of Medicine, Mansoura University, Mansoura, Egypt; 2 Program of Medicine and Surgery, Mansoura National University, Gamasa, Egypt; 3 Department of Medical Parasitology, Faculty of Medicine, New Mansoura University, New Mansoura,, Egypt; 4 Department of Pathology, Faculty of Medicine, Mansoura University, Mansoura, Egypt; 5 Department of Medical Parasitology, Faculty of Medicine, Mansoura University, 2 El Gomhouria Street, Mansoura, 35516 Egypt

**Keywords:** Congenital toxoplasmosis, Thyroid gland, Thyroid hormones, Thyroid antibodies, Rat model

## Abstract

**Purpose:**

The thyroid gland is one of the most vital endocrine organs. It is responsible for the synthesis and secretion of hormones principally triiodothyronine (T3) and thyroxine (T4). These hormones play a significant role in the functions and the metabolism of the body. The thyroid gland could be affected by several infections, among them *Toxoplasma gondii*. Congenital toxoplasmosis took place when the parasite reached the developing fetus and infected any nucleated cells within it. This study assessed the effect of experimental congenital toxoplasmosis on the thyroid gland structure and function.

**Methods:**

We used 34 Wistar rats and allocated them into two groups: normal control group (17 rats) and congenital toxoplasmosis group (17 rats). After euthanasia, the brain and the thyroid gland was assessed through histopathological examination. Thyroid functions examination was performed through measuring the serum levels of T3, thyroxine T4, and thyroid stimulating hormone (TSH). Moreover, serum levels of thyroid antibodies [thyroid-peroxidase antibody (TPO-Ab) and anti-thyroglobulin (TG-Ab)] were examined.

**Results:**

The examination of thyroid tissues of the congenital toxoplasmosis group showed decreased or absent colloid secretion. About 47.1% of follicles showed degeneration with different grades. Parafollicular cells hyperplasia were observed in 23.6% of specimens. The serum concentrations of T3, T4, and TSH were significantly higher in congenital toxoplasmosis group than the control group. The congenital toxoplasmosis group had lower serum concentrations of TPO-Abs than the control group.

**Conclusion:**

These results indicated that congenital *Toxoplasma* infection could result in a central hyperthyroidism state with alteration of thyroid gland structure in offspring.

## Introduction

The thyroid gland is one of the most important organs within the endocrine system. It is the first endocrine organ to develop during intrauterine life [1]. In humans, thyroid gland development starts about four weeks of gestation [2], while in mice and rats, it starts to develop at ninth day and 12th day of gestation respectively [3, 4]. There are numerous similarities of the thyroid gland structure and function among vertebrates. They include the organization of thyroid follicles, thyroid hormones, and their control by pituitary thyrotropin [5].

The predominant thyroid hormones are triiodothyronine (T3) and tetraiodothyronine (thyroxine, T4) [5]. Both hormones are crucial for several physiological functions within the body including the regulation of growth, development, and metabolic rate [6]. Also, they have stimulating effects on proteolysis, lipolysis, glucose absorption, and gluconeogenesis. Moreover, they are essential for the central nervous system and bone maturation in utero and in young age [7].

The synthesis and release of thyroid hormones is controlled by the hypothalamic-pituitary axis. The hypothalamus secretes thyrotropin-releasing hormone (TRH) which stimulates the anterior pituitary to release thyrotropin (thyroid-stimulating hormone [TSH]) [6].

Several infections could lead to thyroid dysfunction. Bacterial infections (such as *Mycobacterium tuberculosis* and *Treponema pallidum*), viral infections (such as Mumps, Rubella, and parvovirus B19), fungal infections (such as *Candida*, *Aspergillus*, and *Histoplasma*), and parasitic infections (such as *Toxoplasma gondii* and *Blastocystis hominis*) were reported as causative agents of thyroid gland dysfunction [8–11].

*Toxoplasma gondii* is a ubiquitous parasite. It had a worldwide distribution, infecting about one-third of the human population worldwide. Its seroprevalence ranged from 10% to more than 90% in some areas [12]. In 80–90% of immunocompetent individuals, toxoplasmosis was asymptomatic, or patients could suffer from flu-like symptoms [13]. In contrast, the disease could be severe or even fatal in patients with compromised immune system. Also, the parasite could vertically pass through the placenta and reach the developing fetus, resulting in congenital toxoplasmosis [14]. Congenital toxoplasmosis prevalence was reported to be between 0.1 and 0.3 per 1000 live births [15, 16].

The relation between thyroid gland and *Toxoplasma gondii* was described in the literature [17, 18]. Several experimental and clinical studies revealed the impact of *Toxoplasma* infection on thyroid hormones. Stahl and Kaneda [19] found decreased serum levels of T4 in *Toxoplasma*-infected mice that was attributed to hypothalamic dysfunction and a disturbance of the pituitary’s pulsatile release of TSH rather than to primary thyroid dysfunction.

Also, there was a remarkable association found between *Toxoplasma* infection and abnormal biochemical thyroid activity in *Toxoplasma*-infected patients as stated by Salman and Mustafa [20]. Furthermore, Eisa et al. [21] observed a significant prevalence of thyroid dysfunction within *Toxoplasma*-seropositive patients. Moreover, the link between this infection and thyroid antibodies was outlined by several researchers. The serum level of anti-*Toxoplasma* IgG antibodies was reported to be significantly higher in patients with autoimmune thyroid diseases (AITDs) than the controls [22]. Latent toxoplasmosis was linked to higher thyroid hormones production and AITDs in pregnant women [23–25]. However, the impact of congenital *Toxoplasma* infection on the thyroid gland hasn’t been established yet. In this work, we studied the effect of congenital *Toxoplasma* infection (using ME49 strain) on the thyroid gland structure and function in a Wister rat model.

## Materials and Methods

### The Parasite

*Toxoplasma gondii*-ME49 (type II) strain was used in this study. It was graciously provided by the Faculty of Medicine, Alexandria University, Egypt.

### The Animals

A total number of 34 rats were included in the study. They were divided as normal control group; composed of 17 Wistar rats (9 males and 8 females) as offspring of normal uninfected mothers and the congenital toxoplasmosis group; composed of 17 Wistar rats (6 males and 11 females) that were congenitally infected by *Toxoplasma* ME49 strain.

The offspring of congenital toxoplasmosis group were obtained by infection of their mothers by 20 *Toxoplasma* ME49 strain cysts [26] by oral gavage at the 12th day of pregnancy [27]. Following birth, the pups of infected rat mothers were separated from the mothers. Instead, *Toxoplasma*-serologically negative foster rat mothers were used to feed the pups to prevent lactogenic transmission of the parasite from the infected mothers. After 21 days of birth, the offspring were weaned and were allocated into new cages according to sex. The rats had free access to food and water. They were housed in well-ventilated cages with appropriate hygienic conditions, natural light/dark cycle, and a temperature of 21 ± 3 °C. After 60 days of birth, offspring rats were sacrificed.

The rats of the congenital toxoplasmosis group were included in the study according to the following: *Toxoplasma* tissue cysts were visualized in their brain homogenates. Also, anti-*Toxoplasma* IgG antibodies were demonstrated and showed positive results in sera of these rats. Those with negative infection status (negative tissue cysts in brain homogenates and negative anti-*Toxoplasma* IgG antibodies results) were excluded from the study.

This study had the approval of the local Institutional Review Board Ethical Committee, Faculty of Medicine, Mansoura University (Code number: MDP.21.02.58).

### Assessment of Serum Anti-Toxoplasma IgG Antibodies

At the time of euthanasia, blood samples were obtained from the offspring of the congenital toxoplasmosis model group. Blood was left undisturbed at room temperature for 10 to 20 min in order to allow it to clot. Centrifugation was used for 20 min at 2,000–3,000 rpm to remove the clot. The infection status of offsprings were evaluated [28] using anti-*Toxoplasma* IgG ELISA kit (Rat anti-*Toxoplasma* antibody IgG (anti-tox IgG) ELISA kit (Catalogue No. In-Ra0939), Wuhan Fine Biotech Co. Ltd).

### Brain Toxoplasma Cyst Visualization and Counting

After euthanasia, the brain of each offspring was removed, and portions of the brains homogenized using tissue homogenizer with one ml saline each. Using a high-power (x40), 10 µl of the brain suspension was counted using a light microscope. Ten high power fields (HPF) were used to count the cysts, and an estimate of the mean number of cysts was made for each offspring [29].

### Histopathological Examination of the Brain and the Thyroid Gland

Brain sections of both rat control group and infected rat offsprings were fixed in neutral buffered formalin 10% and were stained by hematoxylin and eosin staining [30]. The following score was used as follows: Score 0: no inflammation, Score 1: single minimal inflammatory foci, Score 2: widespread minimal inflammation, Score 3: single moderate inflammatory foci, Score 4: widespread moderate or severe inflammatory foci [31].

The thyroid gland of the rats of both infected and control groups were fixed in neutral buffered formalin 10%, processed, and stained by hematoxylin and eosin [30]. The degeneration of thyroid follicles was scored depending on the extent of the disruption of thyroid architecture [32] as follows: Score 0: normal thyroid, Score 1: involvement of less than 25% of the gland, Score 2: between 25% and 50%, Score 3: between 50% and 75%, Score 4: complete architectural disruption. Other pathological findings were graded as the following: Score 0: absent, Score 1: mild, Score 2: moderate, Score 3: severe inflammation [33].

### Assessment of Thyroid Hormones

At the time of euthanasia, blood samples were withdrawn from the rats of both groups. Blood was left undisturbed at room temperature for 10–20 min to allow it to clot. The samples were centrifuged at 2,000–3,000 rpm for 20 min to remove the clot. Serum levels of T3, T4, and TSH were measured [34] using Rat T3 (Triiodothyronine) ELISA kit (Catalogue No. ER1720), Wuhan Fine Biotech Co. Ltd, Rat T4 (Thyroxine) ELISA kit (Catalogue No. ER1721), Wuhan Fine Biotech Co. Ltd, and Rat TSH (Thyroid stimulating hormone) ELISA kit (Catalogue No. ER1411), Wuhan Fine Biotech Co. Ltd respectively.

### Assessment of Anti-Thyroid Antibodies

Serum levels of anti-thyroid antibodies )thyroid-peroxidase antibody [TPO-Ab] and anti-thyroglobulin [TG-Ab]( were estimated [35] using Rat TPO-Ab (anti-Thyroid-Peroxidase Antibody) ELISA kit (Catalogue No. ER1870), Wuhan Fine Biotech Co. Ltd and Rat TG-Ab (Rat anti-Thyroglobulin antibody) ELISA kit (Catalogue No. In-Ra1440), Wuhan Fine Biotech Co. Ltd respectively.

### Statistical Analysis and Data Interpretation

Data analysis was performed by SPSS software (SPSS Inc., PASW statistics for windows version 25. Chicago). Qualitative data were described using numbers and percentages. Quantitative data were described using median (minimum and maximum) for non-normally distributed data after testing normality using Kolmogrov-Smirnov test. The significance of the obtained results was judged at the (≤ 0.05) level. Fisher exact test and Monte Carlo tests were used to compare qualitative data between groups as appropriate. Mann Whitney U and test were used to compare between 2 studied groups for non-normally distributed data. Linear regression was used to assess the effect of combination of more than 2 independent variables on continuous outcome after log transformation.

## Results

### Serum Anti-*Toxoplasma* IgG Antibodies

Anti-*Toxoplasma* IgG antibodies status was assessed according to the absorbance readings and the provided equation in the pamphlet, and rats with positive results (cut-off = 0.215) only was included in the study.

### Brain Tissues *Toxoplasma* Cyst

**Fig. 1:**
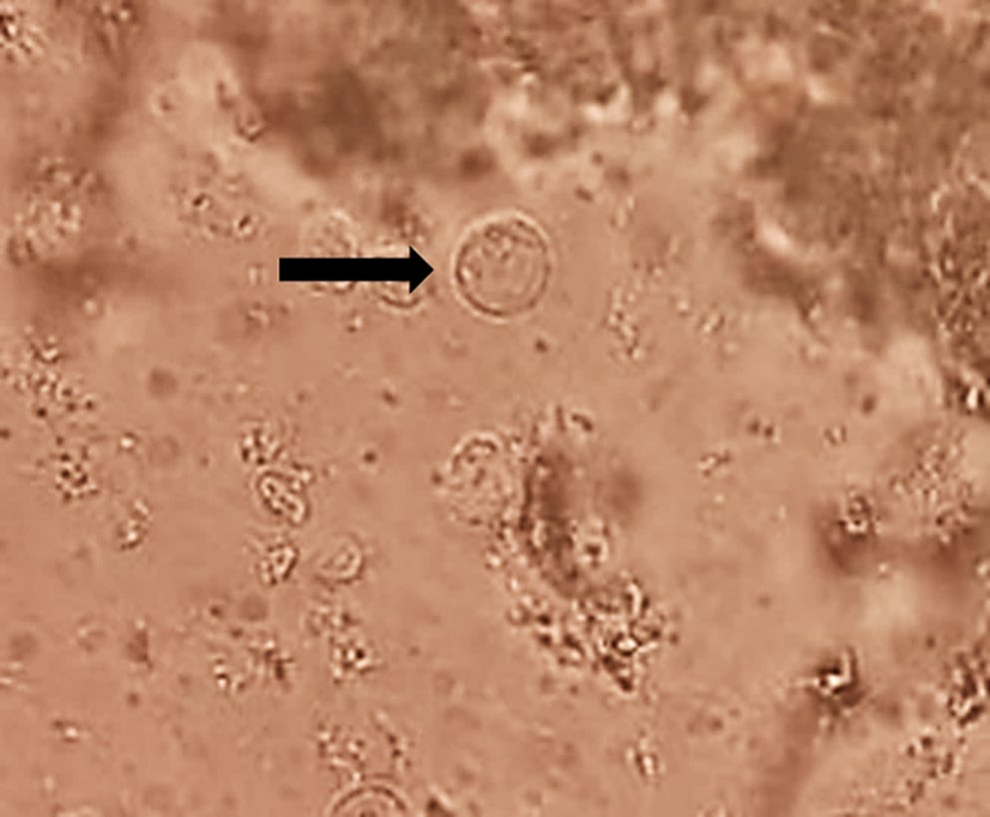
*Toxoplasma* cyst was detected in the brain homogenate of congenital toxoplasmosis-infected rat (x1000)

*Toxoplasma* cysts were demonstrated in brain homogenates of rats of congenital toxoplasmosis group (Fig. 1). The mean *Toxoplasma* cysts count in brain homogenate per 10 HPFs was 6.62 ± 5.41.

### Histopathological Examination of the Brain

**Fig. 2:**
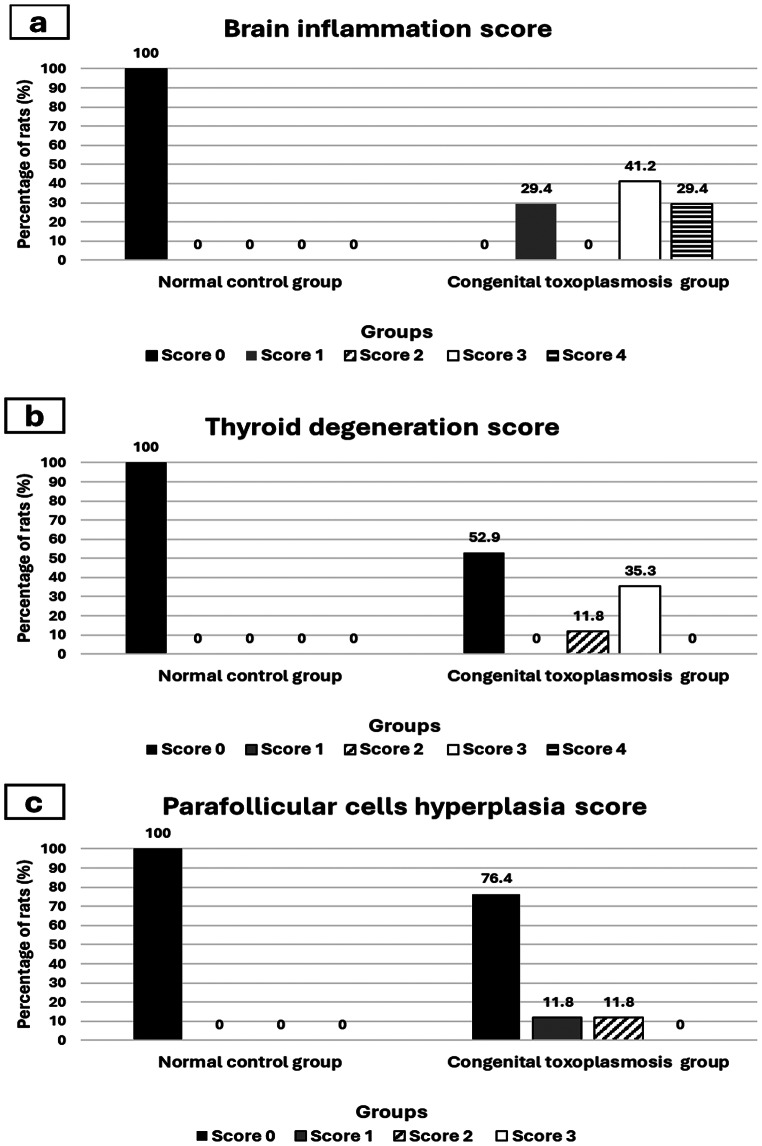
A comparison between the control group and the congenital toxoplasmosis group according to histopathological scores used in the study. (**a**): Brain inflammation score. (**b**): Thyroid gland degeneration score. (**c**): Parafollicular cells hyperplasia scores

**Fig. 3:**
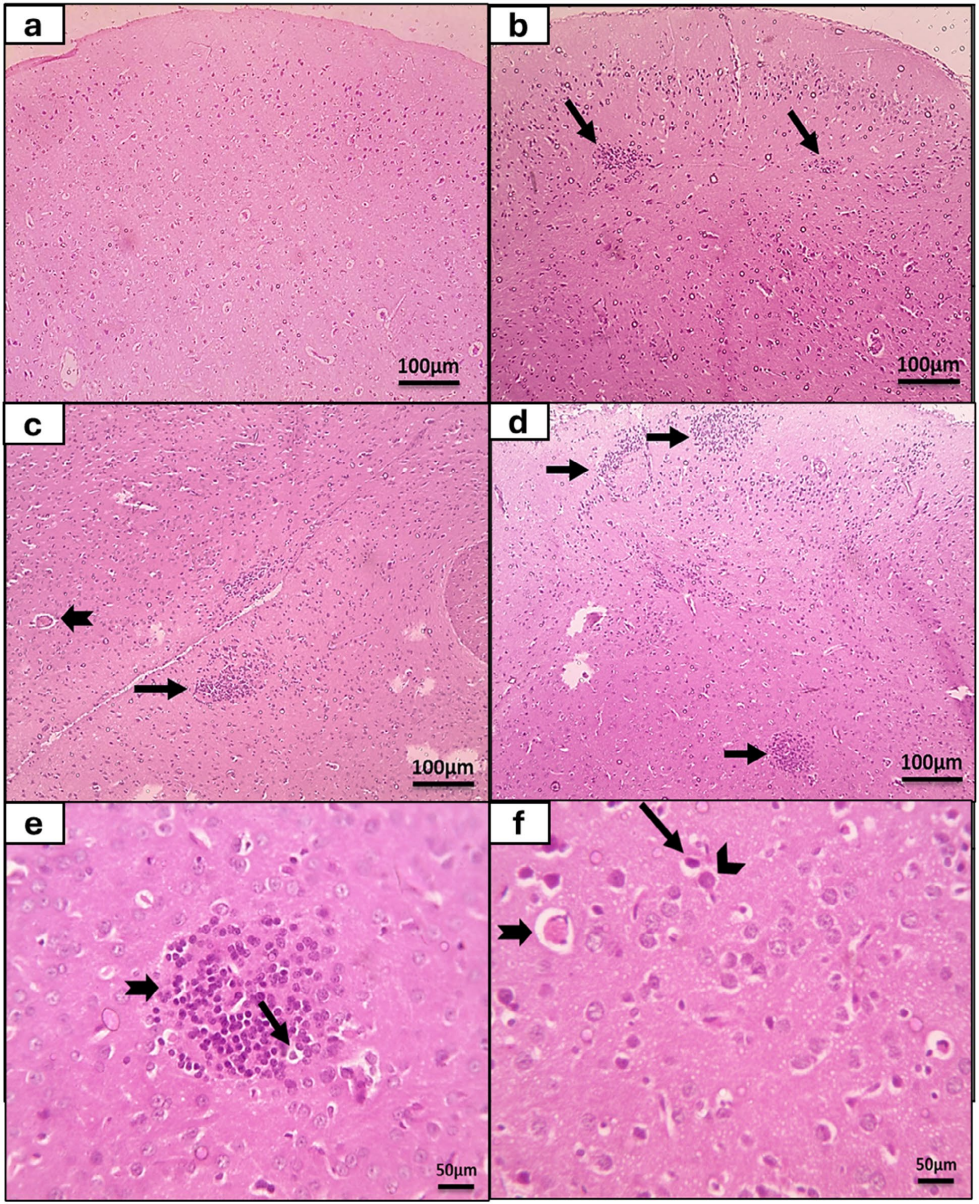
The histopathological studies of the brain of the rat offsprings in the experimental study. (**a**): A brain section (grey matter) of non-infected rat showed normal neurons and glial cells with no evidence of inflammation (score 0) (Haematoxylin and Eosin x 100). (**b-e**): A brain sections (grey matter) of congenital toxoplasmosis-infected rat showed foci of minimal lymphocytic cells infiltrates (black arrow) (score 1) (**b**: H&E×100); single moderate lymphocytic cells infiltrates (thin black arrow) and congested blood vessel (thick black arrow (score 3) (**c**: H&E x 100); multifocal gliosis and widespread moderate lymphocytic cells infiltrates (black arrows) (score 4) (**d**: H&E x 100); focal gliosis with lymphocytic cells infiltrates (thick black arrow) around necrotic neurons (thin black arrow) (**e**: H&E x400), degenerated neurons (thin black arrow), *Toxoplasma* cyst (arrowhead), and congested blood vessel (thick black arrow) (**f**: H&E x400)

According to inflammation and gliosis score (Fig. 2a), 100% of brain tissues of the control group had score 0 with normal neurons and glial cells and no evidence of inflammation (Fig. 3a). While in congenital toxoplasmosis model group, 29.4% of brain tissues showed foci of minimal lymphocytic cells infiltrates and gliosis thus they had score 1 (Fig. 3b). Also, 41.2% of brain tissues attained score 3 with foci of moderate lymphocytes infiltration and gliosis (Fig. 3c), and 29.4% of brain tissues showed score 4 with widespread moderate lymphocytic infiltrates and gliosis (Fig. 3d and e). Moreover, *Toxoplasma* cysts were clearly seen in brain specimens of congenitally infected rats. Furthermore, congested blood vessels and degenerated neurons were demonstrated (Fig. 3f).

### Histopathological Examination of the Thyroid Gland

**Fig. 4:**
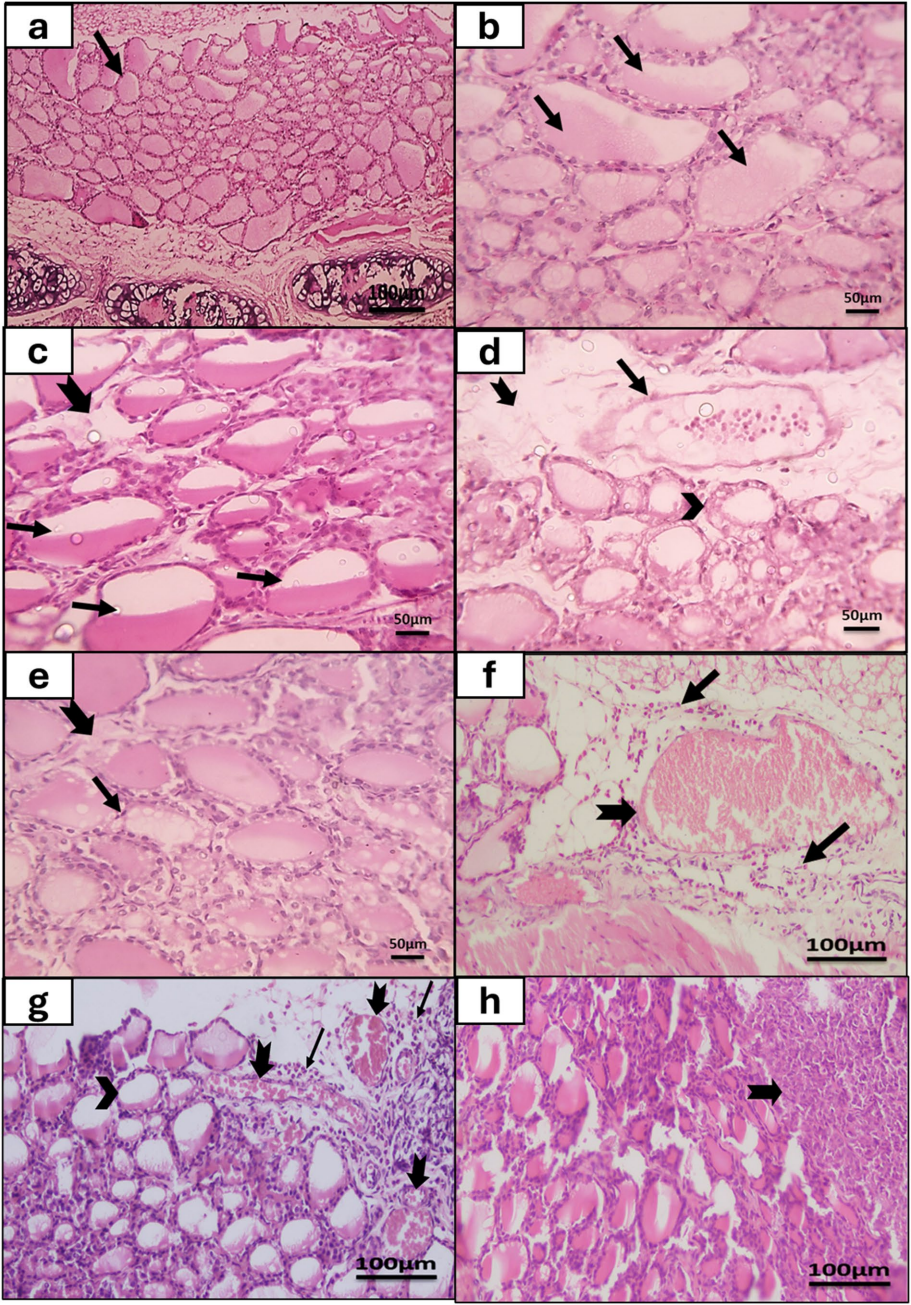
The histopathological studies of the thyroid gland of the rats of the study. (**a**, **b**): A thyroid gland section of normal control rat showing normal follicles with variable size, lined by low cuboidal follicular cells and were filled with eosinophilic colloid (arrow) (**a**: H&E x100; **b**: H&E x400). (**c-h**): thyroid gland sections of *Toxoplasma* congenitally-infected rat, (**c**) the section showed markedly decreased colloid secretion (thin arrow) and mild interstitial oedema (thick arrow) (H&E x400); (**d**) the section showed congested blood vessel (thin arrow) with perivascular oedema (thick arrow), and focally disintegrated follicles lined by vacuolated follicular cells (arrowhead) with decreased or absent colloid secretion (H&E x400); (**e**) the section showed focally decreased colloid secretion with scalloping (thin arrow) with mild interstitial oedema (thick arrow) (H&E x400); (**f & g**) the sections showed multiple congested and dilated intra-thyroid blood vessels (thick arrows) that were surrounded by mixed inflammatory cells infiltrate composed of neutrophils, plasma cells, and lymphocytes (thin arrows), and decreased colloid amount within thyroid follicles (arrow head) (H&E x200); (**h**) the section showed non-caseating epithelioid granulomatous inflammation of the thyroid gland formed of epithelioid histiocytes (thick arrow) (H&E x200)

The thyroid gland of the control group showed normal thyroid architecture with variable-sized thyroid follicles. The follicles were lined with low cuboidal cells and filled with eosinophilic colloid (Fig. 4a and b). No inflammatory cells were detected in all tissue sections of the normal groups.

Meanwhile the thyroid follicles of *Toxoplasma*-congenitally-infected rats showed diminished colloid (Fig. 4c and d, and 4g) or colloid scalloping (Fig. 4e). Also, vacuolated follicular cells lining some follicles (Fig. 4d), congested blood vessels and mild interstitial edema were detected (Fig. 4d and f, and 4g). Mild to moderate inflammatory infiltrates composed of mixed inflammatory cells such as neutrophils, plasma cells, and lymphocytes were seen extravasated from markedly congested intra-thyroid blood vessels (Fig. 4f and g). Also, some thyroid gland sections of infected congenitally-infected rats showed non-caseating epithelioid granulomatous inflammation formed of epithelioid histiocytes (Fig. 4h).

**Fig. 5:**
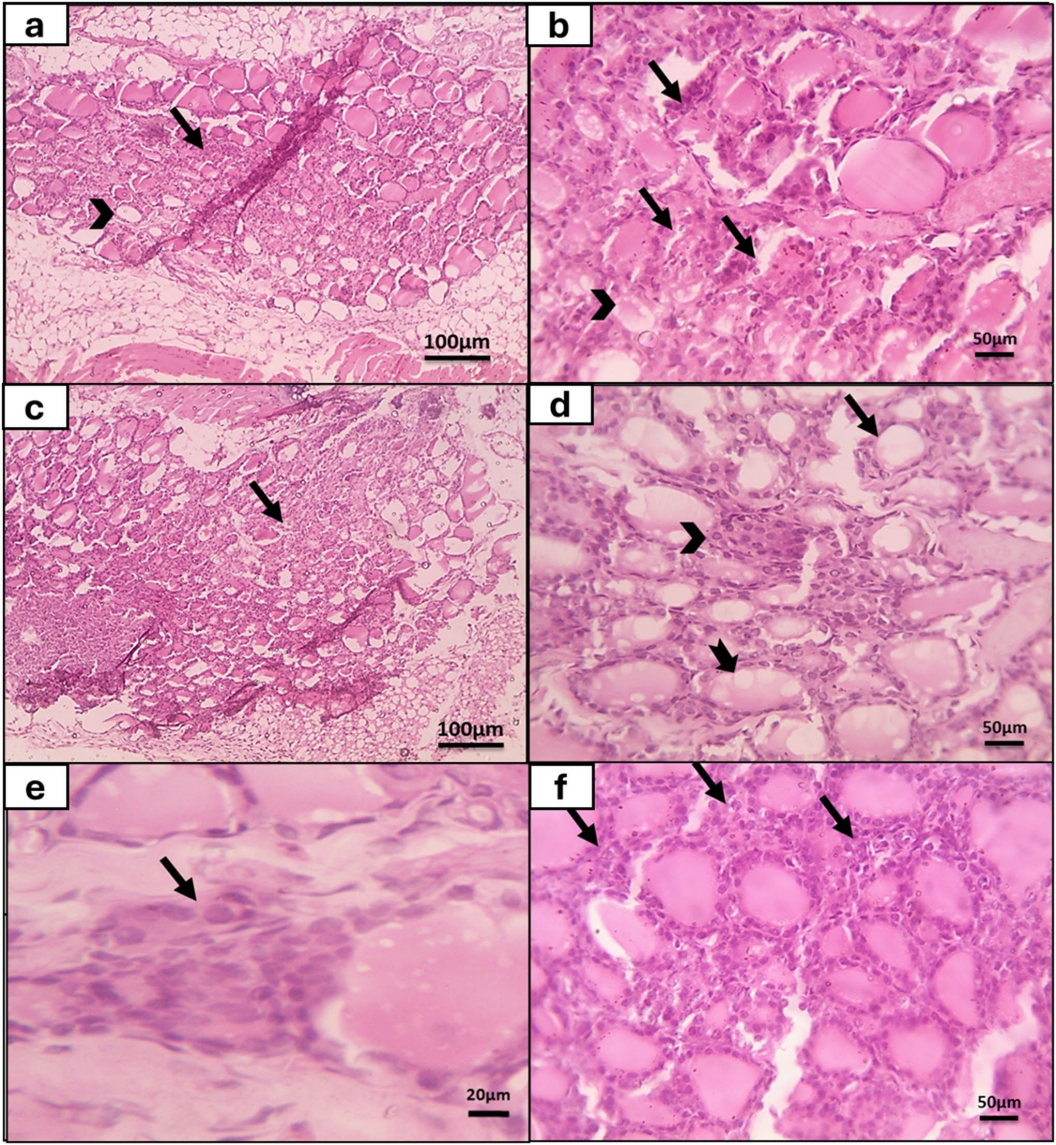
Histopathological examination of the thyroid gland of the congenital toxoplasmosis rat group showed disintegrated follicles (arrow) that involve 25–50% of thyroid gland **(score 2)** and decreased or absent colloid secretion (arrowhead) (**a**: H&E x100); focally disintegrated follicles lined by degenerated or necrotic follicular cells (arrow) and decreased colloid secretion (arrowhead) (**b**: H&E x400); disintegrated follicles (arrow) that involved 50–75% of the gland **(score 3)** (**c**: H&E x100); markedly decreased colloid secretion (thin arrow), vacuolated colloid (thick arrow), and mild focal hyperplasia **(score 1**) of parafollicular cells (arrowhead) (**d**: H&E x400); hyperplasia of parafollicular cells (arrow) (**e**: H&E x1000); moderate hyperplasia **(score 2)** of parafollicular cells (arrows) (**f**: H&E x400)

According to the thyroid gland degeneration score (Fig. 2b), all thyroid gland sections of the control group attained score 0 with no detected degeneration of follicles (Fig. 4a and b). While in the congenital toxoplasmosis model group, 11.8% of sections showed follicles degeneration which involved 25–50% of the gland and obtained score 2 (Fig. 5a and b). Moreover, 35.3% of gland specimens showed follicles degeneration that involved 50–75% of the thyroid gland, gaining score 3 (Fig. 5c). While 52.9% of infected rat thyroid sections gained score 0 as there were no detected degenerated follicles.

As regards parafollicular cells hyperplasia (Fig. 2c), there was no evidence of parafollicular cells hyperplasia in thyroid tissue sections of the normal group (score 0). On the other hand, concerning congenital toxoplasmosis group, 11.8% of specimens attained score 1 with mild parafollicular cells hyperplasia (Fig. 5d and e), furthermore, 11.8% of thyroid tissue specimens obtained score 2 with moderate parafollicular cells hyperplasia (Fig. 5f). While 76.4% of tissue sections showed no parafollicular cells hyperplasia (score 0).

Neither the control group nor the congenital toxoplasmosis model group showed *Toxoplasma* tissue cysts in any examined thyroid tissues. Linear regression analysis showed a statistically significant positive impact of congenital *Toxoplasma* infection on the thyroid gland degeneration score. On the other hand, the same test didn’t show significant effect of congenital *Toxoplasma* infection on the score of parafollicular cells hyperplasia (Table 3).

**Table 1:** A comparison of concentrations of T3, T4 and TSH hormones between normal rat control and congenital toxoplasmosis rat model groups

	Normal control group *N* = 17	Congenital toxoplasmosis group *N* = 17	Test of significance
T3 (ng/ml)	1.23(0.28–2.25)	3.5(1.73–15.8)	Z = 4.71 *P* = 0.001*
T4 (ng/ml)	2.75(1.15–11.3)	5.9(2.6–16.6)	Z = 3.68 *P* = 0.001*
TSH (ng/ml)	7.04(4.6-14.27)	21.3(8.97-82)	Z = 4.75 *P* = 0.001*

T3: Triiodothyronine hormone; T4: Thyroxine hormone; TSH: Thyroid stimulating hormone

Values are expressed as median (min-max). Z: Mann Whitney U test. * Statistically significant

### Assessment of Thyroid Hormones

The concentrations of T3 of rats of the congenital toxoplasmosis group were significantly higher than that of the control group (*P =* 0.001). Moreover, the concentration of T4 of the congenital toxoplasmosis group was shown to be higher than its concentration of the control group with statistically significant difference (*P =* 0.001). Additionally, TSH concentration of the congenital toxoplasmosis group was significantly higher than its concentration of the normal group (*P* = 0.001) (Table 1). There were no significant differences in T3 or T4 or TSH concentrations between males and females of both study groups. Linear regression analysis showed a statistically significant positive impact of congenital *T. gondii* infection on T3, T4 and TSH hormones concentrations level (*P* = 0.001) (Table 3).

**Table 2:** A comparison of the anti-thyroid antibodies; TPO-Ab and TG-Ab concentrations between the normal rat control and the congenital toxoplasmosis rat model groups

	Normal control group *N* = 17	Congenital toxoplasmosis model group *N* = 17	Test of significance
TPO-Ab (IU/ml)	4.09(2.19–5.92)	2.71(1.62–4.98)	Z = 2.68 *P* = 0.007*
TG-Ab (Pg/ml)	1942.0(37-4355)	1053(37-4355)	Z = 1.56 *P* = 0.118

TPO-Ab: anti-thyroid-peroxidase antibodies; TG-Ab: anti-thyroglobulin antibodies

Values are expressed as median (min-max). Z: Mann Whitney U test. * Statistically significant

### Assessment of Anti-Thyroid Antibodies

The concentration of TPO-Abs of the control group was higher than that of the congenital toxoplasmosis group with statistically significant difference (*P* = 0.007) (Table 2). The TG-Abs concentration of the control group was higher than that of the congenital toxoplasmosis group but with no significant difference (*P* = 0.118) (Table 2). Also, there were no significant differences between males and females within both study groups regarding TPO-Ab concentration or TG-Abs concentrations. Moreover, Linear regression analysis showed a statistically significant negative effect of congenital *Toxoplasma* infection on TPO-Abs concentrations (*P* = 0.009) (Table 3).

**Table 3:** Linear regression analysis for prediction of degeneration score of thyroid functions, parafollicular cell hyperplasia, different thyroid hormones concentration and anti-thyroid antibodies concentration in the congenital toxoplasmosis rat model

Model		Unstandardized Coefficients		Standardized Coefficients	t	*P* value
		B	Std. Error	Beta		
**Degeneration score of thyroid tissue**						
	(Constant)	-1.294	0.578		-2.239	0.032*
	*Toxoplasma* infection (+ ve)	1.294	0.362	0.540	3.572	0.001*
**Parafollicular cell hyperplasia**						
	(Constant)	-0.353	0.280		-1.260	0.217
	*Toxoplasma* infection (+ ve)	0.353	0.176	0.339	2.009	0.053
**T3 concentration**						
	(Constant)	-2.877	1.503		-1.914	0.065
	*Toxoplasma* infection (+ ve)	4.055	0.942	0.612	4.304	0.001*
**T4 concentration**						
	(Constant)	-2.413	2.328		-1.037	0.308
	*Toxoplasma* infection (+ ve)	5.536	1.459	0.563	3.794	0.001*
**TSH concentration**						
	(Constant)	-19.364	9.832		-1.969	0.058
	*Toxoplasma* infection (+ ve)	27.031	6.163	0.619	4.386	0.001*
**TPO-Ab concentration**						
	(Constant)	5.304	0.668		7.936	0.001*
	*Toxoplasma* infection (+ ve)	-1.161	0.419	-0.446	-2.771	0.009*

* Statistically significant

## Discussion

*Toxoplasma gondii* is a ubiquitous protozoan parasite. It is widely prevalent in both humans and animals. Congenital toxoplasmosis results due to the vertical passage of the parasites through the placenta. Congenital infection sequalae might be devastating for the fetus and neonate [36].

In this study, obvious pathological alterations were seen in *Toxoplasma*-congenitally infected rats brain tissue sections. The congenital infection of rat offspring was proven by the presence of *Toxoplasma* tissue cysts that were observed together with degenerated neurons. Also, different grades of lymphocytic infiltrates and gliosis were detected. Moreover, congestion and perivascular oedema were observed. These findings came in agreement with previous studies [37–39]. The activation of glial cells and immune cells recruitment are the hallmarks of latent *Toxoplasma* infection [40]. Moreover, intracellular pathogens induced the recruitment of peripheral immune cells that might lead to white matter demyelination and degeneration of neurons [41]. After *T. gondii* infection, glial cells and astrocytes released cytokines like IL-1α, IL-6, TNF-α, IFN-γ, and GM-CSF. This immune reaction was responsible for the persistent inflammation during chronic stage, and it directly affected neurons [42].

According to Ozguner and Sulak [1], the thyroid gland is the first endocrine gland to develop in intrauterine life. To the best of our knowledge, this is the first research to study the impact of congenital toxoplasmosis on the histopathology of the rat thyroid gland. The thyroid gland tissue sections of rats of congenital toxoplasmosis group revealed reduced or absent colloid within thyroid follicles with frequently observed scalloping of colloid. Inflammatory infiltrates extravasating from congested blood vessels and non-caseating epithelioid granulomatous inflammation were observed. In addition, marked interstitial edema was detected. Also, various degrees of thyroid follicles degeneration and parafollicular cells hyperplasia were seen. Vacuolated follicular cells were seen lining some thyroid follicles. However, no *Toxoplasma* cysts were demonstrated within the examined thyroid tissue sections. According to a study conducted by Dubey et al. [17], *Toxoplasma* tissue cysts were seen in the thyroid gland of llama in dessiminted visceral toxoplasmosis. *T. gondii* tissue cysts may develop in any host organ. However, they were mostly detected in the nervous and muscular tissues. This was attributed to the immune-privileged nature of these organs, unlike the thyroid gland [43]. In this study, haematoxylin and eosin-stained tissue sections were used to examine the the thyroid gland structure. So, the future use of immunohistochemistry staining techniques will be of great importance to retreive tissue cysts that couldn’t be seen by the haematoxylin and eosin staining. The observed inflammation in this work could be explained by previous studies which observed that latent *Toxoplasma* infection was linked with increased biomarkers of vascular injury and chronic inflammation including intercellular adhesion molecule 1 (ICAM-1), vascular cell adhesion molecule 1 (VCAM-1), and C-reactive protein. Also, these studies documented increased levels of pro-inflammatory cytokines such as IL-6 and myeloperoxidase. Myeloperoxidase is an enzyme that is released primarily by neutrophils and was involved in immune reaction to the parasite [44, 45]. Vascular injury and inflammation could be induced by the periodic rupture of *Toxoplasma* tissue cysts and the release of bradyzoites that occur spontaneously within intermediate hosts. In immunocompetent hosts, this led to activation of immune system and subsequent clearance of the released parasites [46]. Adhesion molecules (VCAM-1 and ICAM-1) were released into circulation in response to inflammation. They induced the adherence of leukocytes to the vascular endothelium and their transmigration [45]. Dendritic cells, macrophages, and neutrophils responded directly to *Toxoplasma* antigens by releasing IL-12 and TNF-α. IL-12 played an important role in the host immune response against intracellular pathogens such as *Toxoplasma*. Also, IFN-γ that was released by CD4 + and CD8 + T lymphocytes played a significant role in immunity defense against *T. gondii* [47].

According to the stress-coping hypothesis proposed by Lindová et al. [48], toxoplasmosis resulted in impairment of the health, causing long-term stress. This stressful condition was accompanied by elevated oxidative stress represented in reactive oxygen species (ROS) and reactive nitrogen species. ROS broke down the polyunsaturated fatty acids in a process named lipid peroxidation, resulting in the production of hazardous compounds such as malondialdehyde (MDA). Nitric oxide and ROS were significantly generated in tissues throughout the acute stage of toxoplasmosis [49]. Moreover, the serum concentrations of MDA were observed to be significantly higher in patients with latent toxoplasmosis [50, 51]. The thyroid gland was very vulnerable to oxidative stress as documented by Iglesias and Diez [52].

Thyroid follicles degeneration and follicular cells cytoplasmic vacuolations might be attributed to the increased oxidative stress that led to higher rate of apoptosis [53, 54]. Additionally, as stated by Hassanin et al. [55], vascular congestion could be explained by the oxidative stress and its associated lipid peroxidation. They could have impact on vessel wall and result in their dilatation and congestion as recorded in our study.

Regarding the detected parafollicular hyperplasia, a possible relation between parafollicular cells and thyroid gland activity was proposed by Gawad et al. [56]. Besides, it was documented that high levels of TSH might directly regulate parafollicular cells [57]. This came in agreement with our study finding of the elevated TSH levels in rats of the congenital toxoplasmosis group when compared to rats of the control group.

The reduced quantity of colloid within most of the follicles could be due to the stimulatory activity of TSH on the follicular cells to produce more thyroid hormones [56]. Colloid scalloping typically occurred when the follicular cells’ epithelium was more active in synthesizing thyroid hormones. This was due to active ingestion of the colloid by the follicular cells [58].

The aforementioned pathological findings were consistent with the hormonal analysis of this study represented in significantly higher serum levels of TSH, T3, and T4 of rats of the congenital toxoplasmosis group compared to the control group. These results pointed to a central hyperthyroidism that may be brought on by hypothalamic or pituitary dysfunctions. Hyperthyroidism was accompanied by variable levels of oxidative stress and oxidative damage throughout the body [59].

The elevated serum levels of thyroid hormones were consistent with earlier human studies [23, 25, 60, 61]. Also, according to Alvarado-Esquivel et al. [28], there was a negative association between *Toxoplasma* seropositivity and hypothyroidism. On the other hand, Kaňková et al. [23] observed that chronically-infected pregnant mothers with *Toxoplasma* had lower serum levels of TSH. Also, our hormonal assay results disagreed with Shapira et al. [62] and Raissi et al. [63] who concluded that there was no effect of latent toxoplasmosis on the thyroid hormones of infected patients.

The effect of toxoplasmosis on the pituitary was rarely verified in the literature. Cases of *Toxoplasma* infection that were accompanied by pituitary adenoma were observed by Zhang et al. [64]. Also, hyperadrenocorticism due to pituitary disease was described in disseminated toxoplasmosis in cat [65].

The possible central mechanisms that influenced thyroid hormones secretion might be due to the direct impact of *Toxoplasma* tissue cysts on particular brain regions that affected brain function. According to Ihara et al. [66], congenital toxoplasmosis was associated with meningoencephalitis, severe perivascular cuffing affecting the periventricular regions, basal ganglia, and hypothalamic regulatory centers [66].

In this work, the localized brain lesions and the inflammatory infiltration driven by the parasite supported these explanations. Meanwhile indirect mechanisms included the local immune response against *Toxoplasma* infection and its associated cytokines that impacted brain physiology and behavior. Moreover, the chronic activation of immune response of the host could induce stress response that triggered hormonal changes [67].

Another potential mechanism that could explain the effect of *T. gondii* infection on thyroid gland hormones is that the infection could result in higher leptin hormone secretion [68]. Leptin activity involved the activation of the hypothalamic-pituitary-thyroid axis. As described by Flier et al. [69] and Nillni et al. [70], leptin induced both the expression of the proTRH gene and the production of TRH in the paraventricular nucleus within the hypothalamus. Besides, proTRH expression could be stimulated by leptin through an indirect mechanism to increase TRH secretion.

Regarding anti-thyroid antibodies, this study revealed significantly higher TPO-Abs concentrations in the control group compared to the congenital toxoplasmosis group. Additionally, TG-Abs concentrations of the control group were comparable to the congenital toxoplasmosis group. In contrast, other human studies observed elevated titers of thyroid antibodies in patients infected with *Toxoplasma* [24, 61, 71].

These discrepancies between studies results could be attributed to the limited duration of study as Kaňková et al. [23] documented that TPO-Abs increased over time after *Toxoplasma* infection. Moreover, it was proposed that latent infection had a cumulative effect on TPO-Abs concentrations.

The hygiene hypothesis stated that the reduced early-life exposure to infections might raise the risk of autoimmune disorders due to the imbalance between the Th1 and Th2 responses. In contrast to helminths infections that induced Th2 immune response, *Toxoplasma*, as a protozoan infection, was noted to elicit a potent Th1 immune response. *T. gondii* could exert its protective role against AITDs by the increased production of IL-10 with different mechanisms [72]. *Toxoplasma* not only enhanced the production of IFN-γ, but also IL-10 in T-bet + T helper cells. This suggested that the parasite activated regulatory mechanisms during both acute and chronic phases of infection [73].

The activation of FoxP3 + T cells could be an additional immunosuppressive mechanism exerted by *Toxoplasma* infection. Their activation attenuated the immune response throughout latent infection probably by decreasing the availability of the potent proinflammatory IL-2 [74]. In this regard, Jeong et al. [75] demonstrated that IL-10-producing FoxP3 + Tregs and Bregs induced by *Toxoplasma* infection efficiently regulated allergic inflammatory responses. Furthermore, TGF-β participated in the immunosuppression that existed during the latent stage of infection [76].

A recent study conducted by Tipisova et al. [77] described a relation between the elevated concentrations of dopamine and the low levels of TG-Abs. Thus, it was proposed that dopamine inhibited the production of TG-Abs leading to increased thyroid gland activity. The pathogenesis and the development of autoimmune disorders could be impacted by the course of congenital *Toxoplasma* infection; further assessment of thyroid parameters and immune-histochemical studies in the future will be helpful to provide a comprehensive view of how *Toxoplasma* infection influences the thyroid gland.

## Conclusion

Congenital toxoplasmosis resulted in alteration of the thyroid gland structure and functions in experimental rat model. It was represented in thyroid inflammation, follicles degeneration, parafollicular cells hyperplasia, and absent or decreased colloid within follicles. Moreover, the infection resulted in higher serum levels of T3, T4, and TSH compared to control group.

## Data Availability

No datasets were generated or analysed during the current study.
